# Case Report: Fluoxetine-induced leucocytoclastic vasculitis

**DOI:** 10.3389/fpsyt.2026.1803583

**Published:** 2026-03-31

**Authors:** Yanhua Qin, Jiashu Yao, Xiaoyun Jiang, Wei Chen

**Affiliations:** 1 Department of Psychiatry, Sir Run Run Shaw Hospital, School of Medicine, Zhejiang University, Hangzhou, Zhejiang, China; 2 Department of Dermatology, Sir Run Run Shaw Hospital, School of Medicine, Zhejiang University, Hangzhou, Zhejiang, China

**Keywords:** case report, drug adverse reaction, fluoxetine, rash, vasculitis

## Abstract

We report a case of leukocytoclastic vasculitis (LCV) in a 74-year-old woman, occurring seven months after the initiation of fluoxetine therapy for generalized anxiety disorder (GAD). The cutaneous lesions resolved upon discontinuation of fluoxetine but recurred promptly upon rechallenge. Notably, subsequent exposure to paroxetine, another selective serotonin reuptake inhibitor (SSRI) previously tolerated by the patient, also elicited a similar but less severe rash, suggesting cross-sensitization. Transition to venlafaxine, a serotonin-norepinephrine reuptake inhibitor (SNRI), led to complete and sustained resolution of the skin lesions. This case highlights three key clinical points: the potential for delayed-onset cutaneous adverse reactions to SSRIs even after months of uneventful treatment; the occurrence of cross-reactive hypersensitivity among SSRIs; and the importance of considering alternative antidepressant classes with distinct pharmacological profiles in patients with SSRI-associated vasculitis.

## Introduction

Selective serotonin reuptake inhibitors (SSRIs) are first-line agents for major depressive and anxiety disorders due to their favorable efficacy and safety profiles. SSRI-associated cutaneous adverse reactions encompass a broad spectrum, ranging from mild urticaria, petechiae, and photosensitivity to severe conditions such as leukocytoclastic vasculitis (LCV), Stevens-Johnson syndrome, and toxic epidermal necrolysis (1–5). However, the overall incidence of cutaneous reactions to SSRIs remains rare. Fluoxetine, a widely used SSRI, is recommended as first-line pharmacotherapy for major depressive and anxiety disorders. Reported cutaneous adverse reactions to fluoxetine are uncommon and have included urticarial vasculitis, serum sickness, urticaria, ecchymoses, toxic epidermal necrolysis and Stevens-Johnson syndrome (2, 6–10). The onset of SSRI-induced cutaneous lesions typically occurs early in treatment, with a median time of 28 days (range 12–90 days) from initiation (11). We present a case of LCV that developed seven months after starting fluoxetine. To the best of our knowledge, this is the first reported case of fluoxetine-induced LCV, underscoring the possibility of markedly delayed hypersensitivity reactions to this drug class and the necessity for long-term vigilance.

## Case presentation

**Figure 1:**
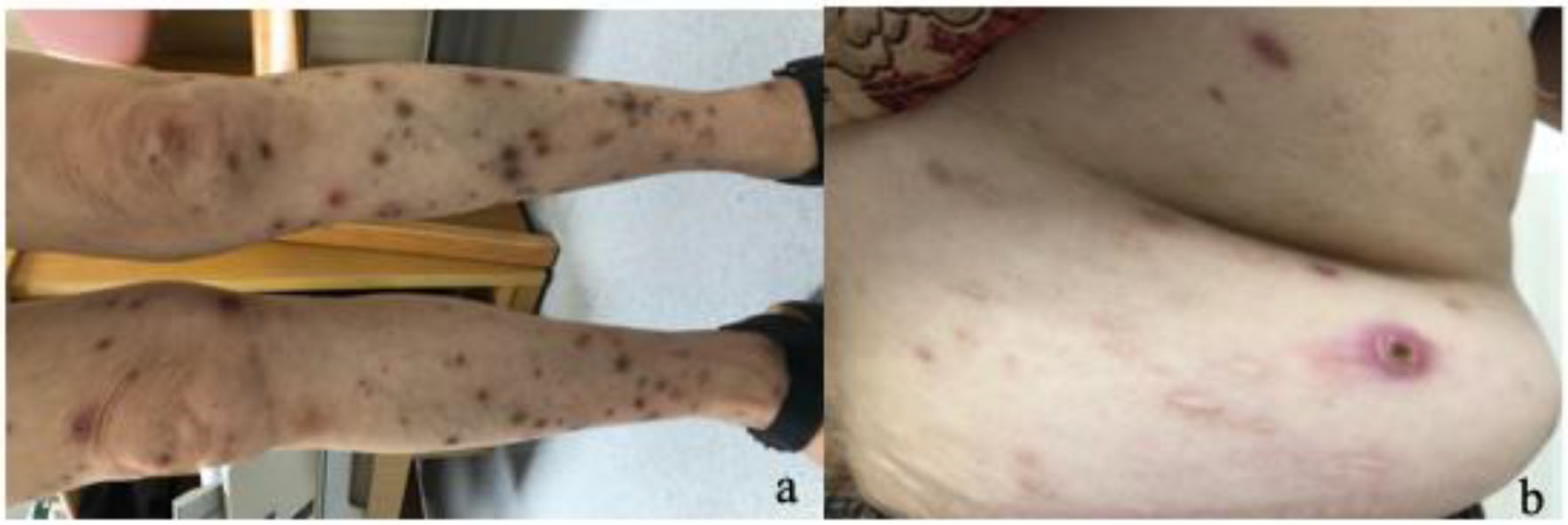
**(A)** Skin lesions, characteristic aspect of diffuse distribution. **(B)** Skin lesions, typical aspect with reddish or pink bumps, necrosis, depressed porcelain-white lesions and white atrophic scars.

**Figure 2:**
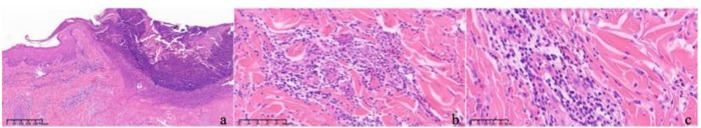
**(A–C)** Skin biopsy revealed epidermal necrotic ulcers, fibrinoid necrosis of vessel walls, and a significant infiltration of lymphocytes and neutrophils in the surrounding dermis and subcutaneous fat layer. Around the vessel wall with signs of nuclear dust, eosinophils, neutrophils and mononuclear cells.

A 74-year-old woman presented with a diffuse skin rash that appeared seven months after initiating fluoxetine hydrochloride dispersible tablets (20 mg daily) for generalized anxiety disorder (GAD). Her psychiatric history included prior treatment with paroxetine (20 mg/day) from 2014 to 2017, which was switched to fluoxetine in June 2017 due to paroxetine withdrawal reactions. She tolerated fluoxetine well for the first seven months (June 2017–January 2018). However, from February 2018 to December 2019, during continued fluoxetine therapy, she developed a non-pruritic, tender, erythematous rash consisting of nodular lesions diffusely distributed over her back, chest, abdomen, and all four extremities, with sparing of the hands and feet (**Figure 1**). A skin biopsy was performed (**Figure 2**), but no specific treatment for the rash was initiated. Upon remission of her GAD, fluoxetine was discontinued from January to March 2020, resulting in complete resolution of the skin lesions within several weeks.

**Figure 3:**
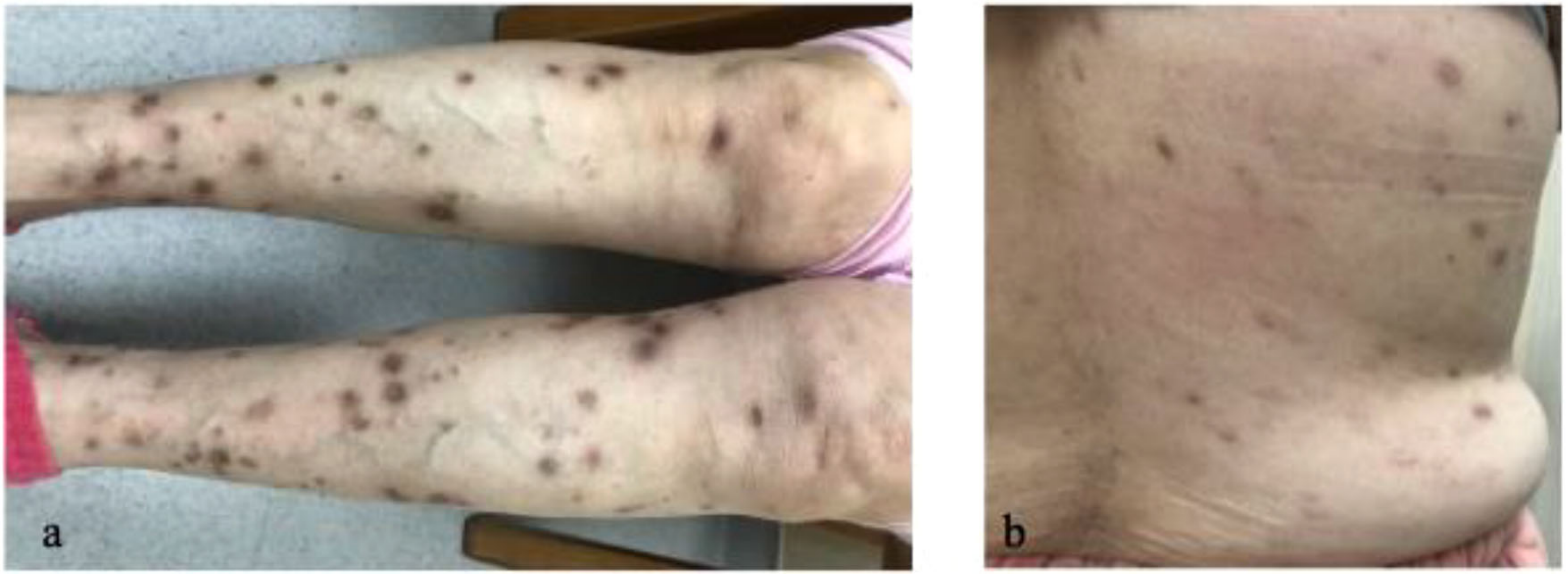
**(A, B)** Resolution of lesions at one-month follow-up after switching to venlafaxine.

In April 2020, due to recurrent anxiety symptoms, fluoxetine was reinitiated. This rechallenge prompted a rapid recurrence of the rash. Subsequently, paroxetine was reintroduced (April 2024 - January 2025), which resulted in attenuated cutaneous manifestations compared to fluoxetine. Finally, on January 13, 2025, her medication was switched to venlafaxine 75 mg daily. The skin lesions resolved spontaneously within one week of starting venlafaxine, and she remained free of new rash with stable GAD control at a one-month follow-up (**Figure 3**).

### Medical history and concurrent medications

The patient had a history of hypertension and coronary atherosclerotic heart disease. Her long-term medications, stable for over 15 years, included metoprolol extended-release 47.5 mg daily, rosuvastatin 5 mg nightly, and enteric-coated aspirin 0.1 g daily. She reported no known drug or food allergies.

### Laboratory data

Complete blood count, C-reactive protein, erythrocyte sedimentation rate, urinalysis, antinuclear antibody, liver and renal function tests, and serology for hepatitis B, hepatitis C, and HIV, were consistently within normal limits or negative.

### Physical examination

Vital signs were normal. Cutaneous examination (**Figures 1A, B**) revealed multiple elevated, firm, non-pruritic, painless papules and nodules (4–7 mm in diameter) diffusely involving the back, chest, abdomen, and all four extremities, with sparing of the hands and feet. The rash progressed from reddish or pink bumps to depressed porcelain-white lesions with a red rim, finally to a white atrophic scar or pigmentation. Both old and new lesions were present. There was no palm, sole, facial or mucous membrane involvement. Physical examination showed no abdominal tenderness, lymphadenopathy, hepatosplenomegaly, or cardiopulmonary abnormalities.

### Histopathological findings

Skin biopsy (**Figures 2A–C**) revealed epidermal necrotic ulceration associated with a dense inflammatory infiltrate composed of lymphocytes and neutrophils. In the adjacent dermis and subcutaneous fat, blood vessel walls exhibited fibrinoid necrosis. Notably, a perivascular infiltrate of neutrophils and mononuclear cells was present, showing evidence of nuclear dust and eosinophils.

## Discussion

**Figure 4:**
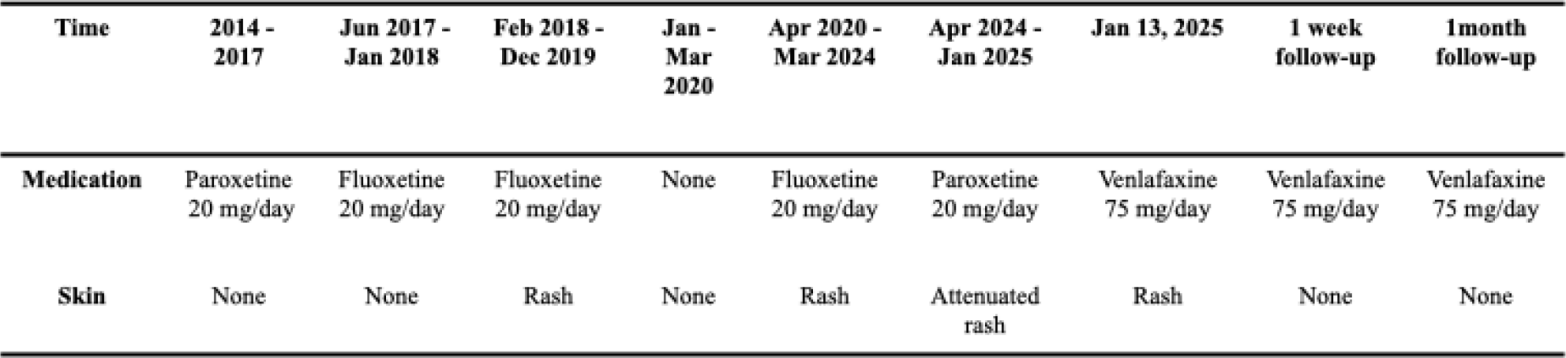
Timeline of medications and rash occurrence.

We attribute the patient’s LCV to fluoxetine based on the strong temporal relationship: the rash appeared after drug initiation, resolved upon its withdrawal, and recurred rapidly upon rechallenge (**Figure 4**). The consistent, long-term use of her other medications (metoprolol, rosuvastatin, aspirin) makes them unlikely culprits. Although the patient was concurrently taking metoprolol, rosuvastatin and aspirin, these medications were less likely to be implicated, given their unchanged regimens before and during the rash episode. Furthermore, thorough investigation ruled out alternative etiologies such as infection, lymphoproliferative disorders, autoimmune diseases, or food allergies, as supported by the absence of systemic symptoms and unremarkable laboratory and physical findings (12). The definitive diagnosis was confirmed by histopathology showing characteristic features of LCV, including neutrophilic infiltration with nuclear dust and fibrinoid necrosis of the vessel walls (13). To the best of our knowledge, based on a comprehensive search of PubMed, Embase, MEDLINE, Ovid, Wiley, Web of Science, and CNKI, this is not only the first reported case of fluoxetine-induced LCV, but also the first to document such a delayed onset-seven months after treatment initiation.

The pathogenesis of LCV involves immune complex deposition in small vessel walls and activation of the complement system. Neutrophil recruitment triggers fibrinoid necrosis of vessel walls, with extravasation of erythrocytes and fibrin. Lymphokines contribute to lesion evolution, and circulating pro-inflammatory cytokines (IL-1, IL-6, IL-8, tumor necrosis factor) are elevated (14). Most drugs are chemically inert and must be metabolized to reactive species prior to haptenation with cellular proteins, and T cells mediate immunity in delayed-type hypersensitivity skin reactions to drugs (15). The pathogenesis of SSRI-induced LCV is unclear, but likely multifactorial. Fluoxetine or its reactive metabolite may act as a hapten, binding to cellular proteins to form immune complexes that deposit in small vessel walls, thereby triggering the typical complement cascade. The seven-month delay in this case is likely due to fluoxetine’s pharmacokinetics. The elimination half-life of fluoxetine is about 1 to 4 days, while that of its metabolite norfluoxetine ranges from 7 to 15 days (16).

The rapid recurrence upon rechallenge is consistent with a drug hypersensitivity mechanism. The notable finding in this case is the subsequent reaction to paroxetine, an SSRI the patient had previously tolerated. This suggests cross-sensitization between fluoxetine and paroxetine, likely due to shared structural or immunological epitopes (17). The patient’s lack of rash during her initial exposure to paroxetine indicates that her sensitivity was not primarily to increased serotonin concentrations (18), but rather to a drug-specific antigenic stimulus triggered by fluoxetine. The successful resolution of the rash with venlafaxine, a serotonin-norepinephrine reuptake inhibitor (SNRI), can be explained by its distinct chemical structure and different pharmacological mechanism, resulting in lower cross-reactivity with SSRIs.

This case is clinically instructive for several reasons. First, the onset of vasculitis seven months after initiating fluoxetine represents a markedly delayed hypersensitivity reaction. Most reported drug-induced cutaneous vasculitis, including the few prior cases of LCV linked to other SSRIs (fluvoxamine, sertraline, escitalopram), occur within five days to six weeks of treatment initiation (19–23). This underscores the imperative for clinicians to consider SSRIs as potential triggers even in patients with long-term, previously uneventful exposure. Second, it provides a clear clinical example of cross-reactive hypersensitivity within the SSRI class following sensitization by one agent. Third, it demonstrates a viable management pathway: switching to an antidepressant from a different pharmacological class (SNRI) after identifying an SSRI-associated adverse event.

Limitations of this case are that patch testing for fluoxetine and human leukocyte antigen gene screening were not performed. This was primarily because the patient declined further testing after symptomatic resolution with drug cessation.

## Conclusion

Although SSRI-induced LCV is rare, clinicians should maintain a high index of suspicion even in patients on long-term therapy. Management of suspected SSRI-induced cutaneous adverse reactions should include: 1) prompt discontinuation of the suspected drug, 2) avoidance of pharmacologically similar drugs within the same class due to the risk of cross-reactivity, and 3) consideration of alternative antidepressants with a different mechanism of action. Increased awareness of delayed hypersensitivity patterns and cross-reactivity among SSRIs is essential for optimal patient management and reducing morbidity.

## Data Availability

The original contributions presented in the study are included in the article/supplementary material. Further inquiries can be directed to the corresponding author.
